# Candidate genes and their alternative splicing may be potential biomarkers of acute myocardial infarction: a study of mouse model

**DOI:** 10.1186/s12872-022-02961-7

**Published:** 2022-11-26

**Authors:** Xuemei Liu, Pengyi He, Ze Zhang, Pengfei Gong, Yunxia Niu, Zhen Bao, Yuchun Yang, Lin Gan, Muhuyati

**Affiliations:** 1 The First Affiliated Hospital of Xinjiang Medical University, Urumqi, China; 2 Faculty of Medicine and Health Sciences, Macquarie University, Sydney, NSW Australia

**Keywords:** Acute myocardial infarction, RNA sequencing, Gene, Alternative splicing, Biomarkers

## Abstract

**Background:**

Acute myocardial infarction (AMI) is one of the leading causes of death in human being, and an effective diagnostic biomarker is still lacking. Whilst some gene association with AMI has been identified by RNA sequencing (RNA-seq), the relationship between alternative splicing and AMI is not clear.

**Methods:**

We retrieved myocardial tissues within 24 h from mice with induced AMI and sham, and analysed the differentially expressed genes (DEGs) and differential alternative splicing genes (DASGs) by RNA-seq. The Gene Ontology (GO) and Kyoto Encyclopedia of Genes and Genomes (KEGG) analysis and protein interaction network analysis were performed on DEGs-DASGs-overlap genes. PCR was used to verify the expression levels of representative genes and alternative splicing in myocardial tissues of AMI and sham mice.

**Results:**

1367 DEGs were identified, including 242 up-regulated and 1125 down-regulated genes, among which there were 42 DASGs. GO analysis showed that the cellular component was primarily enriched in plasma membrane, cell membrane integrity and extracellular region. The molecular function was enriched in protein binding and metal ion binding. The biological process was primarily enriched in cell adhesion, immune system process and cell differentiation. KEGG analysis showed the enrichment was mainly in JAK-STAT and PI3K-AKT signalling pathway. Postn, Fhl1, and Fn1 were low-expressed while Postn alternative splicing was high-expressed in myocardial tissue of AMI mice, which was consistent with sequencing results.

**Conclusions:**

The pathogenesis of AMI involves differentially expressed genes and differential alternative splicing. These differentially expressed genes and their alternative splicing, especially, Fhl1, Fn1 and Postn may become new biomarkers of AMI.

**Supplementary Information:**

The online version contains supplementary material available at 10.1186/s12872-022-02961-7.

## Background

AMI is a life-threatening disease in human being. Thanks to the development of interventional procedures, the incidence and mortality of AMI have gradually declined. However, studies showed that the incidence of AMI among certain population groups, for example females and young people under 40 years of age, had increased again since 2010 [1–3]. Nowadays cardiovascular diseases including AMI are the main causes of death following malignant tumours in developed countries [4]. Currently, an effective predictive diagnostic indicator is still lacking. Therefore, to explore an effective biomarker for AMI has become the focus of biomedical research. In recent years, transcriptome analysis has shown its potential value in the exploration of AMI biomarkers [5, 6]. RNA-seq is one of the important means of transcriptome research and has been widely used in the study of AMI biomarkers [7, 8].

Alternative splicing (AS) is a set of pathways involved in the formation of various protein isoforms from the same gene. The mechanisms that utilised by alternative splicing include alternative splice sites, mutually exclusive exons, exon inclusion/exclusion and the retention of intronic sequences. As an important mechanism of biological regulation of protein’s functional diversity, RNA alternative splicing has attracted increasing attention [9]. Although the concept of alternative splicing has been around for decades, it is only in recent years, along with the advent of high-throughput sequencing methods and new analytical methods, have we begun to understand its significance on a variety of diseases, including cardiovascular disease [10]. However, the relationship between alternative splicing and AMI is still unclear.

In this study, we searched for differentially expressed genes and alternative splicing by RNA-seq in myocardial tissue of AMI mice to explore the relationship between alternative splicing and AMI.

## Methods

### Construction of AMI and sham mouse model

We constructed the mouse model by dividing them into two open-label groups: AMI group and sham operation group (control). A total of thirty-three 8 to10-week-old healthy male C57BL/6 mice were purchased from Huazhong Agricultural University of China and reared under specific pathogens free conditions for 3–7 days, followed by water-fasting for 24 h. All mice were randomly numbered, and then randomly divided into the two groups. Within the groups, the mice underwent the modelling procedures in a random sequence determined by random numbers. In order to establish the AMI mouse model, the mice underwent surgical threading and ligation at the left anterior coronary artery, inducing AMI attacks; whereas the sham mouse model was established by threading only without ligation. Within 24 h after the procedure, 2 living mice from each group were randomly selected, then anaesthetised with 1% pentobarbital sodium administered at 0.12 ml/10 g intravenously and allowed to exsanguinate at the abdominal aorta until death. If this technique failed, the mice were anaesthetised to death by intravenous injection of high-dose pentobarbital sodium (100 mg/kg). The hearts were immediately taken out and frozen at -20 °C for 20 min, then the atriums were removed, and the left ventricles were cut into 1.5–2 mm thickness in parallel with the atrioventricular groove. The slices were put into 2.0% 2,3,5-triphenyl-tetrazolium chloride (TTC, Sigma, USA) for half an hour at 37 °C in darkness, and then fixed with 4.0% paraformaldehyde (Leagene Biotechnology, China) overnight. As a result, normal heart tissue stained red, while the infarct area appeared a pale grey colour. Following the confirmation, all the remaining living mice were sacrificed using the same method, and the myocardial tissues from those mice were retrieved for subsequent analyses. The mice that died prior to exsanguination were not included as we were unable to verify whether or not AMI was successfully induced.

### Construction of RNA-seq library and sequencing

We retrieved 9 individual samples from infarct areas at left ventricles in each group. Within each group, all 9 samples were randomly divided into 3 sets consisting of 3 individual samples each. Then the 3 individual samples in each set were mixed to form a biological repeat sample set. In total, 3 sample sets of AMI mice (AMI1, AMI2, AMI3) and 3 of sham mice (sham1, sham2, sham3) were formed. The sample size was determined based on the principle that the minimum sample size of high-throughput sequencing was 3. Total RNA was extracted with Trizol reagent (Beijing Invitrogen, China). DNA was then removed from the extracted RNA with RQ1 DNase (Promega). To assess the quality and quantity of purified RNA, we measured the absorbance at 260 nm/280 nm (A260/A280) using Nanodroplet One (Thermo). We then used 1.5% agarose gel electrophoresis to further determine the integrity of RNA. In the preparation for RNA-seq, we extracted 1 μg of RNA from each sample, purified the RNA by depleting rRNA using Ribo-off™ rRNA depletion kit (Vazyme, N406-01), then continued to directional RNA-seq library preparation using KAPA Stranded mRNA-Seq Kit for Illumina® Platforms (KK8544). During this process, fragmented mRNAs were converted to double strand cDNA, and then the DNAs were ligated to Diluted Roche Adaptor (KK8726) after end repair and A tailing. We selectively amplified/quantified PCR products corresponding to 300-500bps for sequencing, leaving the strands marked with dUTP (the 2nd cDNA strand) un-amplified, which allowed strand-specific sequencing. Lastly, high-throughput sequencing libraries were prepared as per the manufacturer's manual on Illumina NovaSeq 6000 system for 150 nt paired-end sequencing.

### RNA-seq raw data cleaning and alignment

We firstly discarded raw reads containing more than 2-N bases, then trimmed off adaptors and low-quality bases using FASTX-Toolkit (Version 0.0.13). Any short reads less than 16nt were also discarded. Subsequently, we aligned clean reads to the GRch38 genome by TopHat2 [11] allowing 4 mismatches. Gene reads count was done on uniquely mapped reads and FPKM (fragments per kilobase of transcript per million fragments mapped) was calculated [12].

### Sample correlation analysis

In order to verify the quality of samples after mixing, we conducted correlation analysis on gene expression in AMI1, 2, 3 and sham1, 2, 3 samples.

### DEG analysis

The R Bioconductor package edgeR [13] was utilised to screen out the DEG. A false discovery rate (FDR) < 0.05 and fold change (FC) > 2 or < 0.5 were set as the cut-off criteria for identifying DEG.

### Detection and analysis of alternative splicing

Each sample in RNA-seq data was compared against a specific mapped sequence on the reference genome for AS analysis. TopHat2 was used to analyse the splicing sites of each sample. In AS events (ASE) analysis, one of the annotated transcripts of each gene was selected as the gene model, in other words, the reference transcript. Then the transcript of AS was analysed against the gene model. We conducted an overall analysis and classification of the splicing sites detected by TopHat2, and generated statistical data of various ASE using ABLas, a program independently developed by the test supplier.

### Differential expression analysis of alternative splicing

In order to understand the difference of the same splicing type detected by each gene between AMI and sham, *t*-test was used for ASE difference analysis (A vs B, A represents AMI and B represents sham). The screening criteria for ASE difference was *p* ≤ 0.05, where *t* > 0 was denoted as up, indicating that the occurrence rate of that splicing type was higher in the AMI than in the sham; *t* < 0 was denoted as down, indicating that the occurrence rate of that splicing type was lower in the AMI than in the sham.

### Overlap analysis of DASGs and DEGs

DASGs and DEGs were integrated and analysed to identify genes with significant differences in expression levels and AS levels (DASGs- DEGs-overlap).

### Gene ontology analysis

GO (http://geneontology.org/) is a database that describes the functions of genes and proteins. GO analysis can be divided into three types: Molecular Function, Biological Process and Cellular Component analysis. We analysed the function of genes using GO enrichment analysis. DASGs and DASGs-DEGs-overlap were mapped to each term of GO database respectively, and the number of genes in each term was counted, then the hypergeometric distribution test was used to obtain significantly enriched GO terms against the background of GO annotation of the whole genome. Lastly, we presented the significantly enriched terms for DASGs and DASGs-DEGs-overlap respectively. Should there be more than 10 significantly enriched terms, the top 10 were selected for presentation.

### KEGG analysis

KEGG (http://www.kegg.jp/) is a database developed through genome sequencing and other high-throughput experimental techniques for understanding complex functions of biological systems, for example, cells, organisms and ecosystems. KEGG pathway enrichment analysis was performed on genes to elucidate their functions. DASGs and DASGs-DEGs-overlap were respectively mapped to each pathway of KEGG database, and the number of genes in each pathway was counted, then the KEGG pathways with significant enrichment were identified by using hypergeometric distribution test against the background of KEGG annotation of the whole genome. Similarly, the top 10 pathways were selected for presentation.

### Protein–protein interaction analysis

String10.0, a tool of known and predicted protein interactions based on biological database and network resources, was used to construct Protein–Protein Interaction (PPI) network of 42 proteins encoded by DASGs-DEGs-overlap genes [14]. The "nodes" and "edges" make up the PPI network, with one node representing a protein and one edge representing the interaction between two proteins.

### Validation of representative DASGs-DEGs-overlap genes

We selected three representative genes Fhl1, Fn1 and Postn from 42 DASGs-DEGs-overlap genes. The selection method was based on the FC, expression level, *p* value or FDR and their correlation with AMI. Total RNA was isolated from nine pairs of mouse myocardial tissues using Trizol reagent (Beijing Invitrogen, China) and reversely transcribed into cDNA using the High-Capacity cDNA Reverse Transcription Kit (Applied Biosystems, Foster City, CA). Quantitative real-time polymerase chain reaction (qRT-PCR) was conducted in a Thermal Cycler Dice Real Time System III (TaKaRa) for the detection of Fhl1, Fn1 and Postn. Data were calculated using the 2^−ΔΔCT^ method. The cDNA was amplified by PCR, and the PCR products were verified by electrophoresis with 2% agarose gel and observed by EtBr staining. We used PS software to scan the gray value of the electrophoretic bands to analyse the expression level of Postn alternative splicing semi-quantitatively. β-actin was set as the control. Primers are shown in Table 1.

**Table1:** Primer Information

Gene symbol	Primer name	Primer sequences(5′–3′)	Production(bp)
Fhl1	Fhl1-F1	GAGTACAAGGGCACCGTCTG	70
	Fhl1-R1	CGGTCCCAATGACTTGCTTG	
Fn1	Fn1-F1	TGGCTGTCAGTCAGAGCAAG	85
	Fn1-R1	CCTAGGTAGGTCCGTTCCCA	
Postn	Postn-F1	GGACCTTGTTTGCACCAACC	147
	Postn-R1	CGGGTTCGAATCCCTTTCCA	
Postn	Postn-AS-F1	ACAAACTCCTCTATCCAGC	292 and 211
	Postn-AS-R1	TCTGTCACCGTTTCGCCTTC	
actin	mouse actin F	GGCTGTATTCCCCTCCATCG	154
	mouse actin R	CCAGTTGGTAACAATGCCATGT	

### Statistical analysis

SPSS20.0 was used for statistical analysis. Mean ± standard deviation was used for data measurement, and constituent ratio was used for counting data. Two-tailed Student’s *t*-test was used for the comparison between the two groups, with *p* < 0.05 indicating statistically significant difference (Additional file 1: Table S1).

## Results

### The mouse models were successfully constructed

A total of 28 mice were used in the study, and 22 model mice were successfully constructed, including 11 AMI mice and 11 sham mice respectively. A total of 6 mice died during or soon after modelling procedures but prior to exsanguination, thus excluded from further analyses. TTC staining was performed on the myocardium of the selected mice to confirm a successful modelling, as shown in Additional file 2: Figure S1.

### The quality of sequencing data was high

The quality and quantity of purified RNA for sequencing were high. The A260/A280 value of total RNA in mouse myocardial tissue was between 1.7 and 1.9, and the total amount of extracted RNA was more than 10ug, which was sufficient. The RNA bands of 28srRNA, 18srRNA and 5srRNA were clear, indicating that the integrity of total RNA was good, and all RNA samples were qualified. Database construction and high-throughput sequencing of samples were successfully carried out in this project. Illumina Novaseq 6000 sequencing platform were used for paired-end sequencing on the constructed database, and high-quality transcriptome data were obtained. The effective sequences of all samples accounted for more than 97% of the original sequences. We quantified the percentage of the base ratio with a sequencing error rate of less than 0.1% (Q30) and GC content. The Q30 was more than 94% and the GC contents of all samples were about 50%. In addition, there was no significant deviation in the sequencing results, indicating that our sequencing results had high accuracy and could be used for subsequent detection and analysis.

### Sample correlation was high

The correlation analysis was conducted for each sample and the correlation coefficients were all close to 1, indicating that gene expression of each sample was similar, in other words, data homogeneity was high, and sequencing data quality was good. The results were shown in Additional file 3: Figure S2.

### Detection and analysis of alternative splicing

**Fig. 1:**
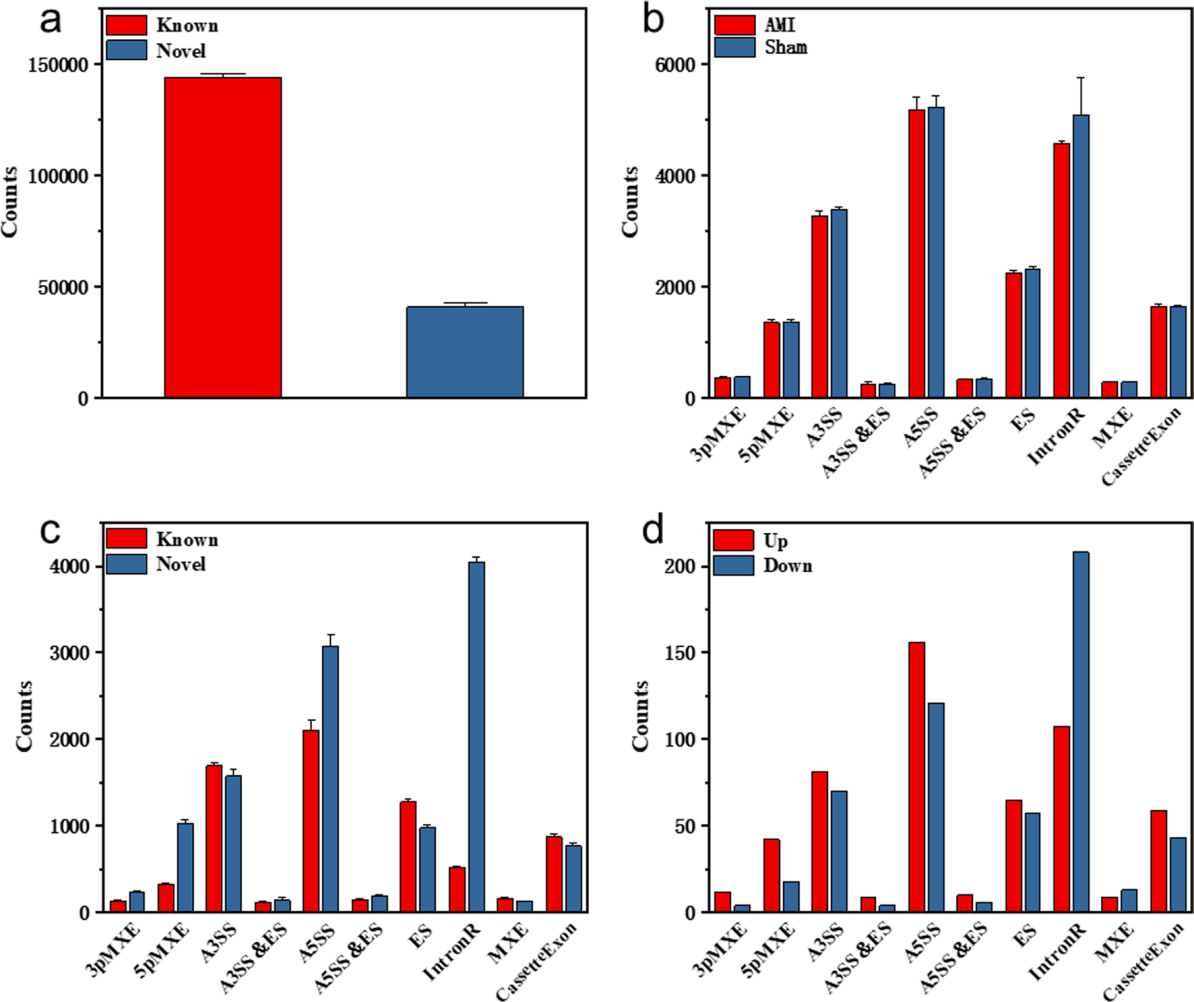
Alternative splicing analysis. **a** The detection of all AS sites; “Known” refers to annotated AS sites; “Novel” refers to new AS sites; **b** The detection of 10 types of alternative splicing in AMI and sham; **c** The distribution of known and novel AS types detected in AMI mice. **d** The distribution difference of all AS types detected in DASGs in AMI and sham groups. “Up” indicates that the expression of AS types in AMI group is higher than that in sham group, and “Down” indicates that the expression of AS types in sham group is higher than that in AMI group. The error bars represent the standard deviation of levels for 3 samples in two separate groups (*n* = 6)

According to the sequencing data, a large number of new splicing sites were detected in myocardial tissues of AMI and sham mice, and the detection levels of known and novel splicing sites in the two groups were similar. We conducted statistical analysis on the known and novel splicing sites in all samples, as shown in Fig. 1a. There were 10 common alternative splicing types, including ES (Exon Skipping), A5SS (Alternative 5 'Splice Site), A3SS (Alternative 3′ Splice Site), IntronR (Intron Retention), MXE (Mutually Exclusive Exons), 5pMXE (Mutually Exclusive 5' UTRs), 3 pmXE (Mad Exclusive 3 'UTRs, Mutually Exclusive 3' UTRs), Cassette exon (Exon), A3SS&ES (Alternative 3 'splice site and Exon Skipping simultaneously) and A5SS&ES (Alternative 5' splice site and Exon Skipping simultaneously). The detection levels of the 10 types of alternative splicing in the two groups were similar, with no significant difference, as shown in Fig. 1b. We classified the 10 types of alternative splicing detected in the two groups by whether they were annotated or not in the genome. Annotated and novel AS types were equally distributed between the two groups. We conducted further statistical analysis on the detection of annotated and novel AS in the 10 types of alternative splicing detected in AMI samples, as shown in Fig. 1c. It could be seen that the new types of alternative splicing detected in AMI were mainly IntronR, A5SS and 5pMXE. *t*-test was used to analyse the difference in the detection levels of the same splicing type detected in each gene in AMI and sham, as shown in Fig. 1d. Among the 10 types of alternative splicing, 550 AS detected in AMI group had higher detection levels than in sham group, and 544 AS detected in sham group had higher detection levels than in AMI group, that is, a total of 1094 detected alternative splicing had different detection levels between the two groups. A5SS, IntronR and A3SS were the most common AS types detected in all genes in AMI group and sham group. The difference was that A5SS was detected mostly in AMI mice, while IntronR was detected mostly in sham mice.

All alternative splices detected in AMI and sham were divided into intron retention (IR) and non-intron retention (NIR) according to different splicing methods. There were 33,262 NIR, accounting for 72.1%, of which 9,960 were annotated and 23,302 were unannotated. There were 12,851 IR, accounting for 27.9%, including 713 annotated and 12,138 unannotated, as shown in Additional file 4: Figure S3.

### 42 DASGs-DEGs-overlap genes were detected

**Fig. 2:**
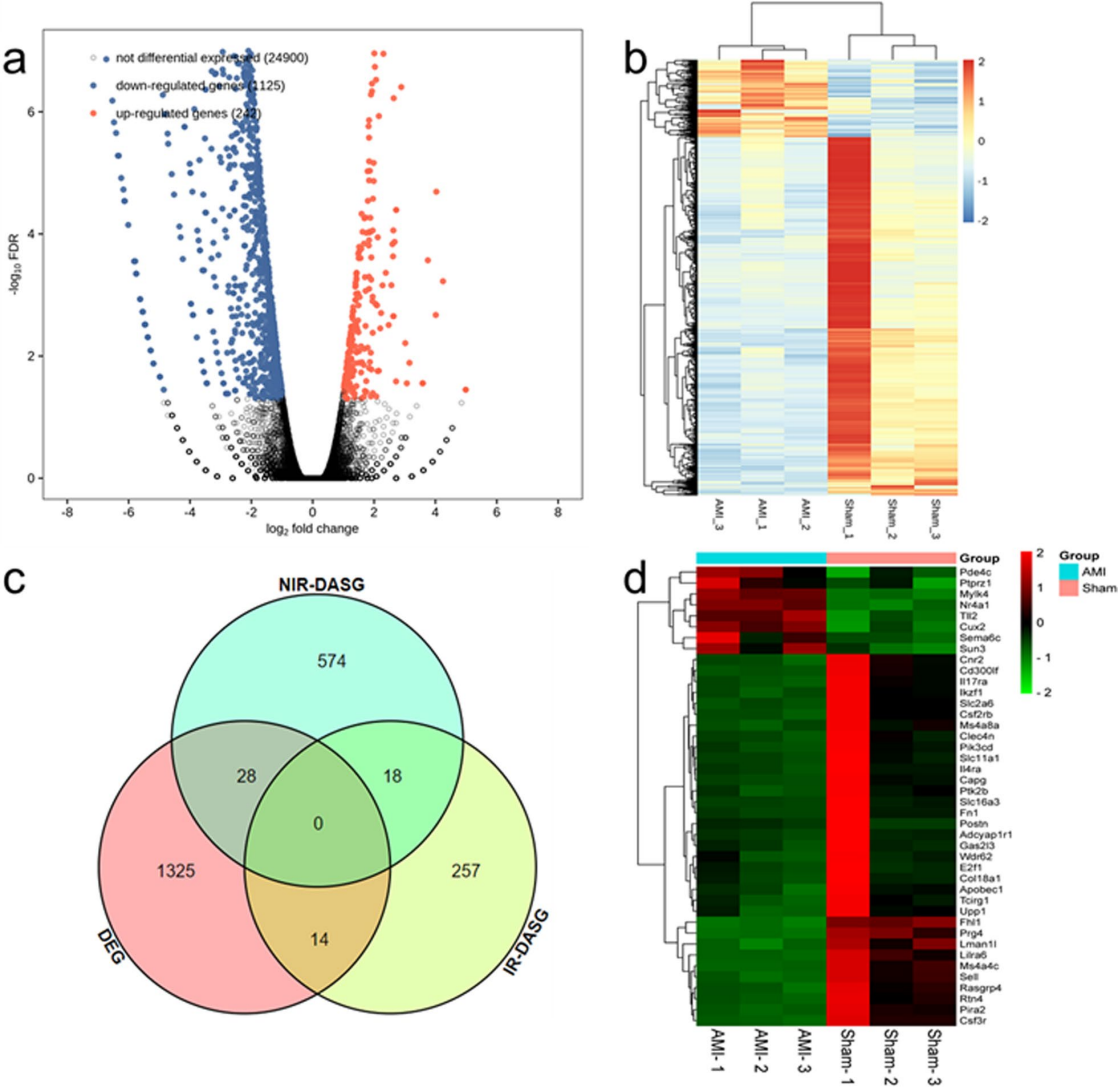
Analysis of DEGs, DASGs-DEGs-overlap. **a** The volcano map of DEGs, the abscissa represents the multiple of change in gene expression levels (log value based on 2), and the ordinate represents the significance of difference in gene expression levels (log value based on 10). Red dots represent significantly up-regulated genes, blue dots represent significantly down-regulated genes, and black dots represent genes with no significant change in expression; **b** The heat map of DEGs; **c** The Venn diagram of DEGs and DASGs overlap analysis, IR is intron retention and NIR is non-intron retention; **d** The heat map of DASGs-DEGs-overlap genes

According to RNA-seq transcriptomic data, a total of 26,267 genes were detected, including 1,367 DEGs, 242 up-regulated and 1125 down-regulated, as shown in Fig. 2a. Cluster analysis was conducted for DEG in the two groups of samples, as shown in Fig. 2b, indicating significant differences in expression patterns of DEGs between the two groups. A total of 909 DASGs were detected in all genes, including 620 DASGs with NIR splicing and 289 DASGs with IR splicing. According to the overlap analysis of DASGs and DEGs, we identified 42 DASGs-DEGs-overlap genes, as shown in Fig. 2c. The information of the 42 genes are shown in Additional file 1: Table S1. Cluster analysis of these 42 overlapping genes between AMI and sham groups showed that the expression patterns of 42 genes in the two groups were significantly different, as shown in Fig. 2d. We further analysed the difference in the expression levels of 9 genes with the highest background expression levels among the 42 genes (Col18a1, Fhl1, Fn1, Mylk4, Il4ra, Postn, Rtn4, Capg and Nr4a1) in myocardial tissues of AMI and sham mice, as shown in Additional file 5: Figure S4. The results showed that Col18a1, Fhl1, Fn1, Il4ra, Postn, Rtn4 and Capg had low expression in AMI and high expression in sham, whilst Nr4a1 and Mylk4 had high expression in AMI and low expression in sham.

### Gene ontology analysis

**Fig. 3:**
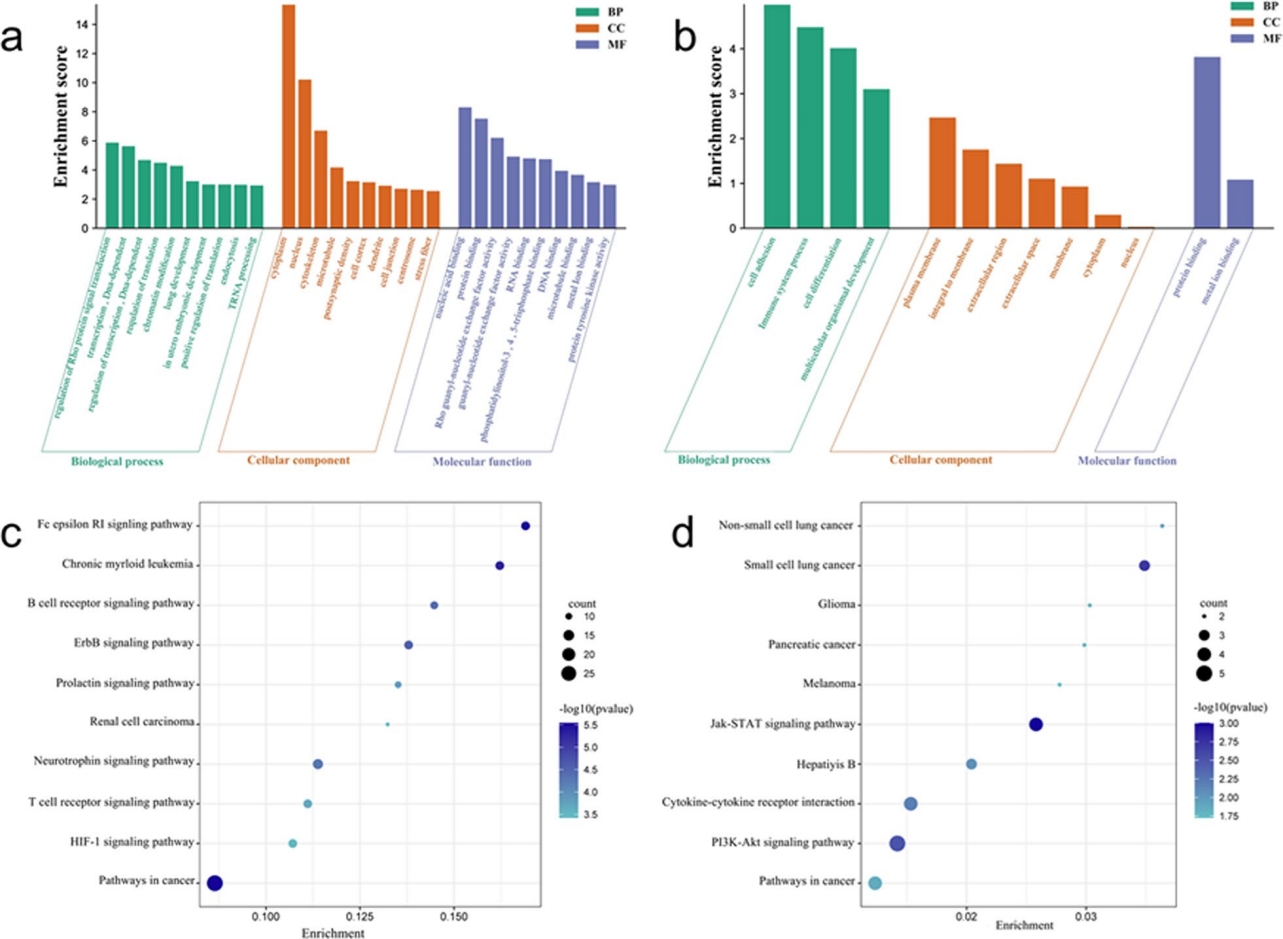
Analysis of GO and KEGG. **a** GO analysis histogram of DASGs; **b** GO analysis bar chart of DASGs-DEGs-overlap genes; **c** KEGG analysis bubble chart of DASGs; **d** KEGG analysis bubble chart of DASGs-DEGs-overlap genes

GO analysis of DASGs showed that Cytoplasm, Nucleus, Cytoskeleton, Microtubule, Postsynaptic density, Cell cortex, Dendrite, Cell junction, Centrosome and Stress fibre were the top 10 enriched cellular components. The top 10 enriched molecular functions were Nucleic acid binding, Protein binding, Rho guanyl-nucleotide exchange factor activity, Guanyl-nucleotide exchange factor activity, RNA binding, Phosphatidylinositol-3,4, 5-trisphosphate binding, DNA binding, Microtubule binding, Metal ion binding and Protein tyrosine kinase activity. The top 10 biological processes enriched were Regulation of Rho protein signal transduction, Transcription, DNA-dependent, Regulation of transcription, DNA-dependent, Regulation of transcription, Chromatin modification, Lung development, In utero embryonic development, Positive regulation of translation, Endocytosis, and tRNA processing, as shown in Fig. 3a.

GO analysis results of DASGs-DEGs-overlap showed that cell components were enriched in Plasma membrane, Integral to membrane, Extracellular region, Extracellular space, Membrane, Cytoplasm and nucleus. Molecular functions were enriched in Protein binding and Metal ion binding. Biological processes were enriched in Cell adhesion, Immune process, Cell differentiation and Multicellular organismal development, as shown in Fig. 3b.

### KEGG analysis

KEGG pathway analysis of DASGs showed that the top 10 enriched pathways were Pathway in cancer, Fc epsilon RI signalling, Chronic myeloid leukemia, ErbB signalling, B cell receptor signalling, Neurotrophin signalling, Prolactin signalling, T cell receptor signalling, HIF-1 signalling and Renal cell carcinoma, as shown in Fig. 3c.

KEGG Pathway analysis of DASGs-DEGs-overlap showed that the top 10 were JAK-STAT signalling pathway, Small cell lung cancer, PI3K-AKT signalling pathway, Cytokin-cytokin receptor interaction, Hepatitis B, Non-small cell lung cancer, Pathway in cancer, Glioma, Pancreatic cancer and Melanoma, as shown in Fig. 3d.

### Protein–protein interaction

7 proteins had the most interaction with others, namely Fn1, Sell, Ptk2b, Pik3cd, Il4ra, Csf2rb and Ikzf1. The proteins with the most pivotal significance were Fn1 and Pik3cd, as shown in Additional file 6: Figure S5.

### The validation results were consistent with RNA-seq results

**Fig. 4:**
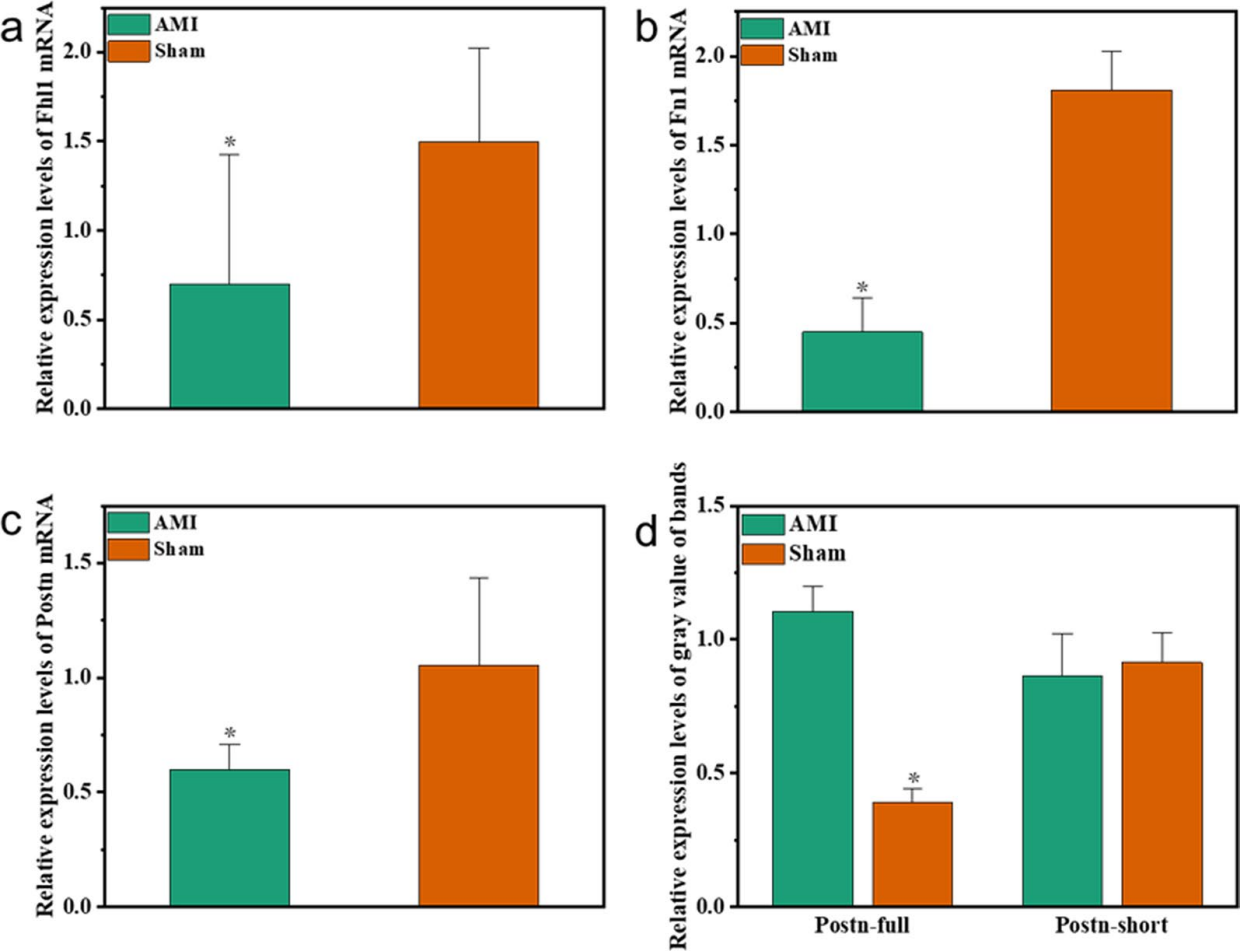
PCR validation results. **a–c** The mRNA expression levels of Fhl1, Fn1 and Postn in AMI and sham groups, respectively; **d** The expression levels of gray value of Postn gene (Postn-short) and its alternative splicing (Postn-full) in PCR electrophoresis bands in AMI and sham. *means *p* < 0.05. The error bars represent the standard deviation of expressions for 6 samples in two separate groups (*n* = 12)

RNA-seq results showed that Fhl1, Fn1 and Postn were all low-expressed in myocardial tissues of AMI mice and high- expressed in myocardial tissues of sham mice. qRT-PCR was used to detect the expression levels of Fhl1, Fn1 and Postn in myocardial tissues of AMI and sham mice. The expressions of Fhl1, Fn1 and Postn were low in AMI mice and high in sham mice, with statistically significant differences between the two groups. The results are shown in Fig. 4a, b, c. qRT-PCR and RNA-seq results were consistent. After literature review, we found that Postn was the most closely related gene among Fhl1, Fn1 and Postn with AMI, therefore Postn was selected as the target gene for subsequent studies. According to RNA-seq results, Postn underwent alternative splicing through cassette exon, and its alternative splicing transcript (ENSMUST00000117373.5) was high-expressed in AMI myocardium, contrary to the Postn gene expression trend. Primers were designed with Postn alternative splicing transcript sequence (long fragment, Postn-full) and reference sequence (short fragment, Postn-short) obtained by sequencing. PCR was used to amplify the alternative splicing product in AMI and sham mouse myocardial tissues. The electrophoretic bands were then semi-quantitatively detected, and the results were consistent with those obtained by sequencing, that is to say, Postn-full was high-expressed in AMI myocardium and low-expressed in sham mouse myocardium. The *p* value between the two groups was 0.001, and the difference was statistically significant, as shown in Fig. 4d.

## Discussion

AMI is a disease with high morbidity and mortality with a trend of affecting younger population. How to prevent, make early diagnosis, improve prognosis and reduce mortality is the direction of biomedical research at present.

Our AMI mouse model was constructed by ligation of the left anterior coronary artery. The mice were sacrificed within 24 h, and the myocardial tissue was collected for RNA-seq. Through analysis of DEGs and DASE in AMI and sham mice, a total of 1367 DEGs were found, including 242 up-regulated genes and 1125 down-regulated genes. DASE occurred in 42 genes. These results suggested these 42 genes and their AS were correlated with the occurrence and progression of AMI.

From the 42 DASGs-DEGs-overlap genes, we selected Fn1, Fhl1 and Postn for qRT-PCR validation based on the gene expression levels in samples, FC value and correlation with cardiovascular diseases. The validation results were consistent with the sequencing results. This suggests that Fn1, Fhl1 and Postn may be associated with AMI, and further studies are needed to reveal their relationship. We also found that Postn-full splicing was different between AMI and sham mice, and further research is needed to explore the mechanism of Postn-full splicing in AMI.

Fn1(fibronectin) plays an important role in cell adhesion and migration, including embryo formation, healing, coagulation response and host defence, and is one of the components of extracellular matrix proteins [15]. Balashanmugam et al. found through microarray data analysis and protein mapping analysis that Fn1 expression was down-regulated in coronary artery disease (CAD), and was correlated with the gender of CAD patients [16]. Fn1 polymorphisms were found to be associated with vascular disease in a 10-year prospective cohort study in Finland. Huang et al*.* proposed that Fn1, as the target gene of Mir-144-3p and Mir-9-3p and one of the key genes of miRNA-mRNA network, was associated with dilated cardiomyopathy, and the role of Fn1 in dilated cardiomyopathy was mainly in extracellular matrix remodelling [17]. Yuan et al. found that in the infarcted myocardium, the expression level of Mir-144-3p increased; furthermore, decreased expression level of Mir-144-3p was accompanied by the decrease in the fibrosis related gene mRNA and related protein levels [18], suggesting that Fn1 was related to myocardial infarction. There are very few studies on Fn1 in acute myocardial infarction. Our study found that Fn1 was low-expressed in AMI mice, and its LogFC value was high (-2.461) and *p* value was low (< 0.001), suggesting that Fn1 was closely related to the occurrence of AMI. Further qRT-PCR validation was consistent with the sequencing results, suggesting that Fn1 may have an important association with AMI.

Fhl1 is a member of the Fhl (four-and-a-half LIM) family and is found to be expressed in a variety of tissues, including muscle, heart, kidney, lung, brain and ovary, but mainly in skeletal muscle and cardiac muscle. Studies conducted over 2 decades ago found that various isomers of Fhl1 protein, containing different numbers of LIM domains, could be generated by alternative splicing and perform different biological functions [19–21]. Danos et al. found that ifhl1 expression increased in hypertrophic obstructive cardiomyopathy (HCM) after Fhl1 alternative splicing, and HCM significantly deteriorated after Fhl1 ablation, including increased left ventricular hypertrophy, fibrosis and induced pathological remodelling of molecular markers. It was proposed that stress-induced Fhl1 and regulatory molecules that change Fhl1 transcription were the genetic modifications in HCM and played a beneficial role in HCM [22]. Chen et al. found that Fhl1 expression was significantly up-regulated in porcine atrial fibrillation model, and Fhl1 played an important regulatory role in cardiac remodelling through transcriptional regulation and myofilament assembly [23]. Kwapiszewska et al. studied pulmonary arterial hypertension (PH) rats and found that Fhl1 was highly expressed in vascular smooth muscle cells of PH rats. Direct comparison of Fhl1 expression in lung slices of normoxic and hypoxic mice showed that Fhl1 expression was enhanced in pulmonary vessels of hypoxic mice. In addition, Fhl1 was up-regulated in primary pulmonary artery smooth muscle cells (PASMCs) in patients with idiopathic pulmonary hypertension, suggesting that Fhl1 might be an important participant in vascular remodelling, but had no significant effect in cell apoptosis [24]. Our study found that Fhl1 was significantly lower in AMI mice, and differential expression of AS (ENSMUSG00000023092.14, *p* < 0.001) was detected between AMI and sham. Combined with evidences from previous studies, we could suggest that Fhl1 might be a protective protein of normal myocardium. Both Fhl1 and its alternative splicing play important roles in maintaining smooth muscle cell function and avoiding myocardial remodelling. Given that Fhl1 and its alternative splicing have no effect on apoptosis, how they dynamically change after the occurrence of AMI, and whether they can promote cell proliferation still needs further study.

Postn (periostin) is a cell-associated protein involved in cell survival, proliferation, tumourigenesis and inflammatory response, and also participates in extracellular matrix (ECM) responses. Studies have confirmed that Postn plays an important role in cardiogenesis and in coronary artery disease, hypertension, valvular disease and cardiac fibrosis [25–27]. Postn functions by activating integrin-related P38 /MAPK, FAK, PI3K-AKT and Wnt/β-catenin signalling pathways in fibroblasts and vascular smooth muscle cells during cardiogenesis and cardiovascular diseases [28–30]. Previous studies have confirmed that Postn is associated with myocardial infarction, which is consistent with our findings. The very first research on Postn in cardiac diseases indeed started after the discovery of its role in myocardial remodelling in mice post myocardial infarction [31]. Oka et al. subsequently found that Postn promoted scarring and reduced the probability of myocardial rupture in mouse models of myocardial remodelling post myocardial infarction. However, as the mice grew older, spontaneous myocardial fibrosis and hypertrophy appeared, aggravating myocardial remodelling [32]. Onur et al. also found that the absence of Postn ( +) myofibroblasts reduced collagen production and scar formation after myocardial infarction [33]. These findings have suggested that Postn might increase scar formation and myocardial remodelling post myocardial infarction. The human Postn gene forms seven splicing isomers including full-length Postn through alternative splicing between exons 17 and 21 [34]. Balbi et al. proposed in their latest study that exosomes secreted by human explant-derived cardiac progenitor cells (CPC) promoted cardiomyocyte re-entry cycle and proliferation via Postn subtypes expressed on their surface, while recombinant full-length Postn did not yield the same results [35]. This was also confirmed in another study with rats, in which the gene expression levels of four types of Postn including full-length (i.e., full-length PN-1, PN-2-deficient exon 17, PN-3-deficient exon 21, and PN-4-deficient exon 17 and 21) peaked 5–7 days after myocardial infarction in rats. Selective suppression of PN-1 with neutralising antibodies against Postn exon 17 but not PN-2/3/4 resulted in reduced infarction size and scar formation, and prevented ventricular dilatation without affecting myocardial cell proliferation [36]. These studies have suggested that Postn spliceosome is associated with myocardial infarction and can promote myocardial cell proliferation. This correlation is consistent with the findings in this study, where differentially expressed Postn and its alternative splicing were detected within 24 h after myocardial infarction in mice, suggesting that Postn alternative splicing played an important role in the early stage of AMI. In addition, Postn alternative splicing was high in the AMI. Combined with previous research results, we speculated that Postn alternative splicing had protective effects on normal myocardium, and further research would be needed to explore the mechanism of Postn in the occurrence and progression of AMI and its dynamic evolution in the course of AMI. We expected that Postn alternative splicing could be a good candidate for new AMI biomarkers.

We conducted KEGG enrichment analysis on 42 DASGs-DEGs-overlaps and found that they were mainly enriched in JAK-STAT and PI3K-AKT signalling pathways. JAK-STAT is a major signalling pathway for many cytokines and growth factors. JAK-STAT plays a key role in cytokine signal transduction, which is directly responsible for the transfer of stimulus signals to the nucleus and promoting gene transcription. This pathway is widely involved in cellular stress response, apoptosis, inflammation and other biological processes, and plays a key role in the pathogenesis of many cardiovascular diseases [37]. Previous studies have shown that JAK-STAT is related to myocardial apoptosis after AMI [38], and it is closely related to myocardial hypertrophy and myocardial remodelling after myocardial infarction [39]. In this study, the JAK-STAT signalling pathway was the most enriched in the 42 DEGs of alternative splicing, suggesting that the alternative splicing of these 42 genes might be involved through JAK-STAT in the onset and progression of AMI. The PI3K-AKT signalling pathway is another widely confirmed pathway associated with the prevention of cardiac hypertrophy and apoptosis [40, 41]. Previous studies have proved that Postn plays a role in promoting heart muscle and valve regeneration by activating PI3K-AKT [29, 42]. We speculated that Postn alternative splicing could also play an important role in the pathogenesis of AMI by activating PI3K-AKT, which needed to be confirmed by further studies. As seen from our results, DEGs of alternative splicing between AMI and sham mice were mainly enriched in JAK-STAT and PI3K-AKT, the two well-studied pathways related to myocardial cell apoptosis and myocardial remodelling, suggesting the possible involvement of gene alternative splicing in the AMI via these two pathways. These results have provided a new direction for future exploration on the mechanism of AMI.

## Conclusions

The pathogenesis of AMI involves differentially expressed genes and differential alternative splicing. These differentially expressed genes and their alternative splicing, especially, Fhl1, Fn1 and Postn may become new biomarkers of AMI.

## Supplementary Information

**Additional file 1**. **Table S1**: The information of the 42 DASGs-DEGs-overlap genes.

**Additional file 2**. **Figure S1**: Model mice and isolated hearts.

**Additional file 3**. **Figure S2**: Sample correlation coefficient analysis.

**Additional file 4**. **Figure S3**: The proportion diagram of splicing methods.

**Additional file 5**. **Figure S4**: Differences in expression levels of DASGs-DEGs-overlap between the two groups.

**Additional file 6**. **Figure S5**: Protein-Protein interaction (PPI) network.

## Data Availability

The raw sequence data reported in this paper have been deposited in the Genome Sequence Archive [43] in National Genomics Data Center [44], China National Center for Bioinformation/Beijing Institute of Genomics, Chinese Academy of Sciences (GSA: CRA008659) that are publicly accessible at https://ngdc.cncb.ac.cn/gsa.

## Abbreviations

AMI: Acute myocardial infarction
AS: Alternative splicing
ASE: Alternative splicing event
CAD: Coronary artery disease
CPC: Cardiac explants are derived from progenitor cell
DEG: Differentially expressed gene
DASG: Differential alternative splicing gene
ECM: Extracellular matrix
FDR: False discovery rates
FC: Fold change
GO: Gene Ontology
KEGG: Kyoto Encyclopedia of Genes and Genomes
PPI: Protein–protein interaction
PH: Pulmonary arterial hypertension
PASMC: Pulmonary arterial smooth muscle cell
qRT-PCR: Quantitative real-time polymerase chain reaction
RNA-seq: Ribonucleic acid sequencing
