# Supplementary material for: Candidate genes and their alternative splicing may be potential biomarkers of acute myocardial infarction: a study of mouse model
Source: BMC Cardiovasc Disord. 2022 Nov 26;22:505. doi: 10.1186/s12872-022-02961-7 (PMC9701406; doi:10.1186/s12872-022-02961-7)
Supplement: Supplementary file 1 — Additional file 1. Table S1: The information of the 42 DASGs-DEGs-overlap genes. [file 12872_2022_2961_MOESM1_ESM.docx]

**Table S1** The information of DASGs-DEGs-overlap

| **Symbol** | **Gene** | **Chromosome** | **logFC** | **P value** | **FDR** |
| --- | --- | --- | --- | --- | --- |
| E2f1 | ENSMUSG00000027490.15 | chr2 | -1.570 | ＜0.001 | ＜0.001 |
| Ptk2b | ENSMUSG00000059456.11 | chr14 | -1.244 | ＜0.001 | 0.004 |
| Slc11a1 | ENSMUSG00000026177.9 | chr1 | -1.441 | ＜0.001 | ＜0.001 |
| Lilra6 | ENSMUSG00000030427.15 | chr7 | -2.466 | ＜0.001 | ＜0.001 |
| Apobec1 | ENSMUSG00000040613.12 | chr6 | -1.285 | ＜0.001 | 0.003 |
| Tcirg1 | ENSMUSG00000001750.13 | chr19 | -1.254 | ＜0.001 | 0.004 |
| Sema6c | ENSMUSG00000038777.17 | chr3 | 1.037 | 0.002 | 0.040 |
| Pde4c | ENSMUSG00000031842.11 | chr8 | 1.100 | 0.001 | 0.022 |
| Slc16a3 | ENSMUSG00000025161.14 | chr11 | -3.057 | ＜0.001 | ＜0.001 |
| Slc2a6 | ENSMUSG00000036067.10 | chr2 | -1.918 | ＜0.001 | ＜0.001 |
| Col18a1 | ENSMUSG00000001435.12 | chr10 | -1.330 | ＜0.001 | 0.001 |
| Cd300lf | ENSMUSG00000047798.13 | chr11 | -5.823 | ＜0.001 | ＜0.001 |
| Adcyap1r1 | ENSMUSG00000029778.10 | chr6 | -1.060 | 0.001 | 0.024 |
| Ms4a4c | ENSMUSG00000024675.17 | chr19 | -4.024 | ＜0.001 | ＜0.001 |
| Tll2 | ENSMUSG00000025013.12 | chr19 | 1.295 | ＜0.001 | 0.007 |
| Fhl1 | ENSMUSG00000023092.14 | chrX | -1.209 | ＜0.001 | 0.005 |
| Fn1 | ENSMUSG00000026193.13 | chr1 | -2.461 | ＜0.001 | ＜0.001 |
| Pik3cd | ENSMUSG00000039936.16 | chr4 | -1.463 | ＜0.001 | ＜0.001 |
| Upp1 | ENSMUSG00000020407.11 | chr11 | -1.149 | ＜0.001 | 0.014 |
| Cux2 | ENSMUSG00000042589.16 | chr5 | 1.230 | ＜0.001 | 0.005 |
| Cnr2 | ENSMUSG00000062585.9 | chr4 | -1.341 | ＜0.001 | 0.005 |
| Csf2rb | ENSMUSG00000071713.4 | chr15 | -2.683 | ＜0.001 | ＜0.001 |
| Pira2 | ENSMUSG00000089942.7 | chr7 | -2.834 | ＜0.001 | ＜0.001 |
| Ptprz1 | ENSMUSG00000068748.5 | chr6 | 1.185 | 0.002 | 0.034 |
| Ms4a8a | ENSMUSG00000024730.7 | chr19 | -2.719 | ＜0.001 | ＜0.001 |
| Mylk4 | ENSMUSG00000044951.11 | chr13 | 1.900 | ＜0.001 | ＜0.001 |
| Il4ra | ENSMUSG00000030748.8 | chr7 | -1.589 | ＜0.001 | ＜0.001 |
| Rasgrp4 | ENSMUSG00000030589.13 | chr7 | -1.193 | ＜0.001 | 0.012 |
| Gas2l3 | ENSMUSG00000074802.8 | chr10 | -1.350 | ＜0.001 | 0.002 |
| Wdr62 | ENSMUSG00000037020.14 | chr7 | -1.231 | ＜0.001 | 0.012 |
| Sell | ENSMUSG00000026581.12 | chr1 | -2.667 | ＜0.001 | ＜0.001 |
| Prg4 | ENSMUSG00000006014.14 | chr1 | -2.239 | ＜0.001 | ＜0.001 |
| Clec4n | ENSMUSG00000023349.12 | chr6 | -2.311 | ＜0.001 | ＜0.001 |
| Il17ra | ENSMUSG00000002897.3 | chr6 | -2.115 | ＜0.001 | ＜0.001 |
| Postn | ENSMUSG00000027750.14 | chr3 | -1.346 | ＜0.001 | 0.001 |
| Rtn4 | ENSMUSG00000020458.14 | chr11 | -1.498 | ＜0.001 | ＜0.001 |
| Ikzf1 | ENSMUSG00000018654.15 | chr11 | -1.765 | ＜0.001 | ＜0.001 |
| Lman1l | ENSMUSG00000056271.11 | chr9 | -2.689 | ＜0.001 | ＜0.001 |
| Capg | ENSMUSG00000056737.12 | chr6 | -1.830 | ＜0.001 | ＜0.001 |
| Sun3 | ENSMUSG00000040985.11 | chr11 | 2.114 | ＜0.001 | 0.018 |
| Csf3r | ENSMUSG00000028859.12 | chr4 | -3.810 | ＜0.001 | ＜0.001 |
| Nr4a1 | ENSMUSG00000023034.6 | chr15 | 2.009 | ＜0.001 | ＜0.001 |
