# Supplementary material for: Candidate genes and their alternative splicing may be potential biomarkers of acute myocardial infarction: a study of mouse model
Source: BMC Cardiovasc Disord. 2022 Nov 26;22:505. doi: 10.1186/s12872-022-02961-7 (PMC9701406; doi:10.1186/s12872-022-02961-7)
Supplement: Supplementary file 2 — Additional file 2. Figure S1: Model mice and isolated hearts. [file 12872_2022_2961_MOESM2_ESM.docx]

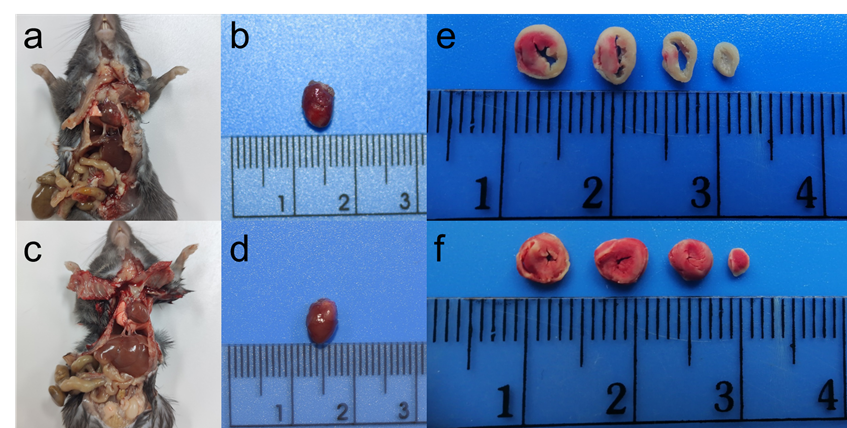


**Figure S1** Model mice and isolated hearts

(a) the dissected AMI mouse; (b) the isolated heart of the AMI mouse; (c) the dissected sham mouse; (d) the isolated heart of the sham mouse; (e) TTC staining of AMI mouse heart; (f) TTC staining of sham mouse heart.
