# Supplementary figures and images for: Candidate genes and their alternative splicing may be potential biomarkers of acute myocardial infarction: a study of mouse model
Source: BMC Cardiovasc Disord. 2022 Nov 26;22:505. doi: 10.1186/s12872-022-02961-7 (PMC9701406; doi:10.1186/s12872-022-02961-7)

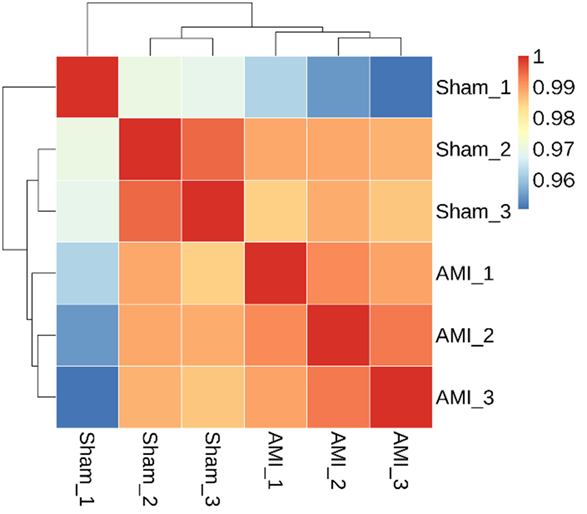


**Figure S2** Sample correlation coefficient analysis

Supplement: Supplementary file 3 — Additional file 3. Figure S2: Sample correlation coefficient analysis. [file 12872_2022_2961_MOESM3_ESM.docx]

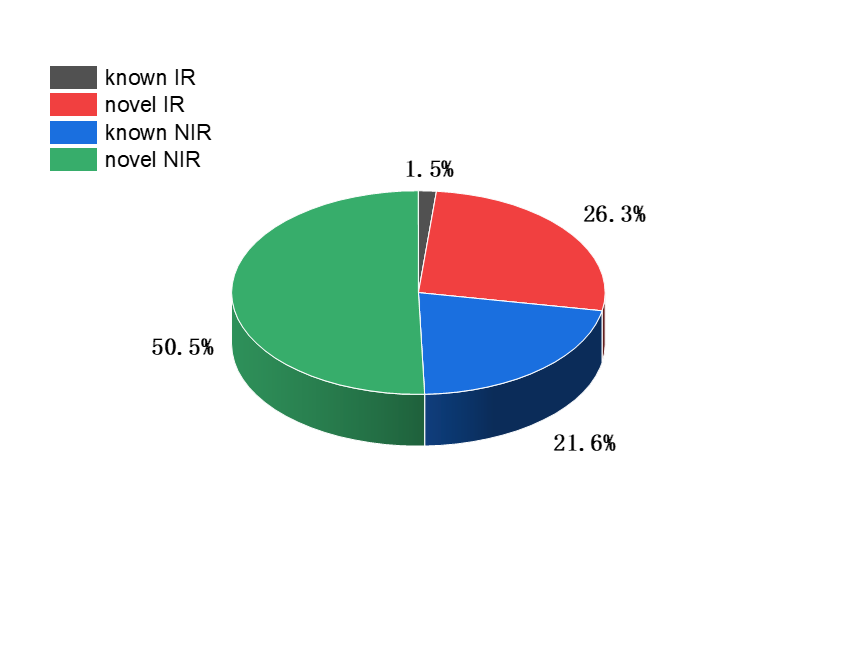


**Figure S3** The proportion diagram of splicing methods

Supplement: Supplementary file 4 — Additional file 4. Figure S3: The proportion diagram of splicing methods. [file 12872_2022_2961_MOESM4_ESM.docx]
