# Supplementary material for: Candidate genes and their alternative splicing may be potential biomarkers of acute myocardial infarction: a study of mouse model
Source: BMC Cardiovasc Disord. 2022 Nov 26;22:505. doi: 10.1186/s12872-022-02961-7 (PMC9701406; doi:10.1186/s12872-022-02961-7)
Supplement: Supplementary file 5 — Additional file 5. Figure S4: Differences in expression levels of DASGs-DEGs-overlap between the two groups. [file 12872_2022_2961_MOESM5_ESM.docx]

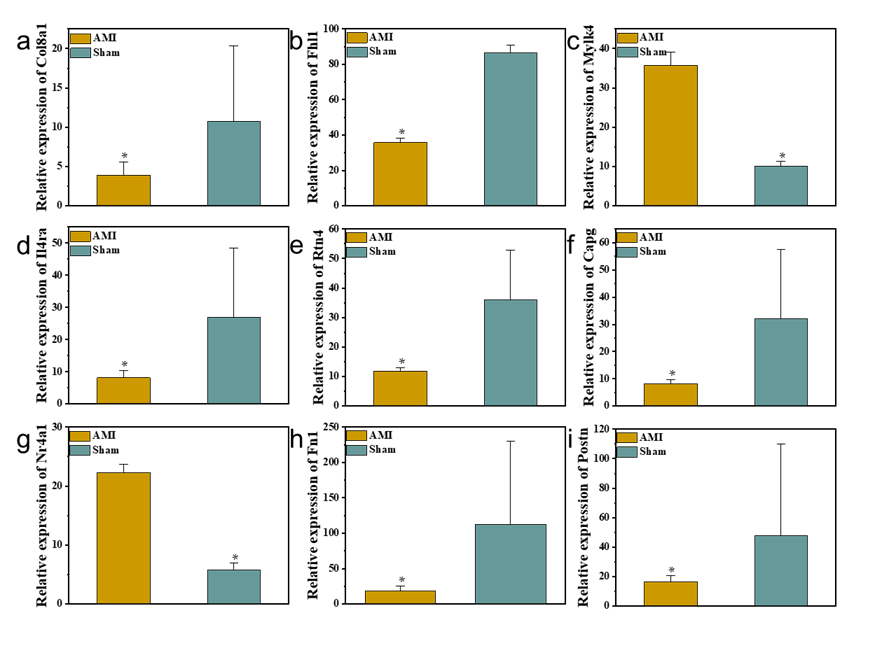


**Figure S4** Differences in expression levels of DASGs-DEGs-overlap between the two groups

(a)-(i) The expression level of 9 genes with high background expression level among the 42 DASGs-DEGs-overlap between AMI and sham. The ordinate is the FPKM value of genes, * means P < 0.05. The error bars represent the standard deviation of expressions for 3 samples in two separate groups (*n*=6).
