# Supplementary material for: Candidate genes and their alternative splicing may be potential biomarkers of acute myocardial infarction: a study of mouse model
Source: BMC Cardiovasc Disord. 2022 Nov 26;22:505. doi: 10.1186/s12872-022-02961-7 (PMC9701406; doi:10.1186/s12872-022-02961-7)
Supplement: Supplementary file 6 — Additional file 6. Figure S5: Protein-Protein interaction (PPI) network. [file 12872_2022_2961_MOESM6_ESM.docx]

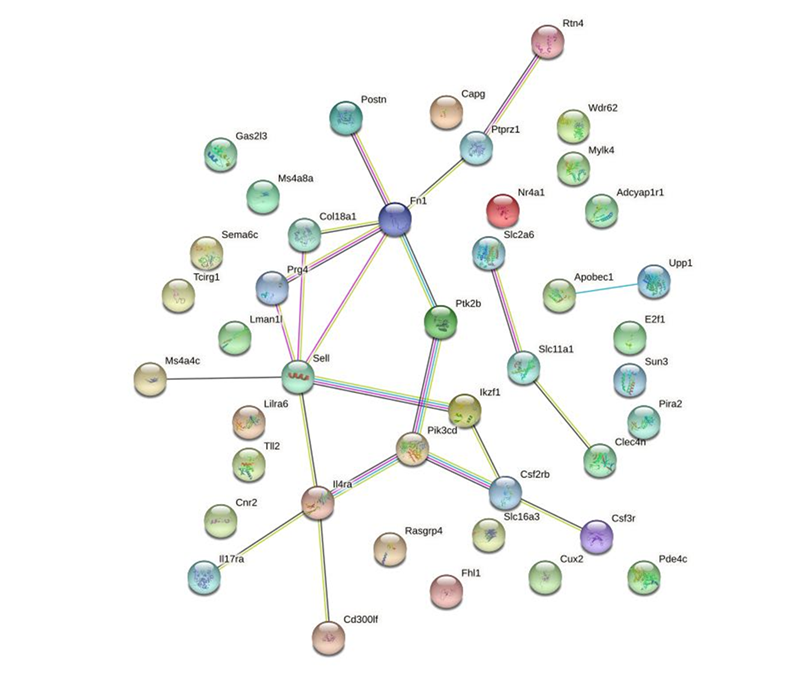


**Figure S5** Protein-Protein interaction (PPI) network

The "nodes" and "edges" make up the PPI network, with one node representing a protein and one edge representing the interaction between two proteins.
